# Irrigation Scheduling Based on Wireless Sensors Output and Soil-Water Characteristic Curve in Two Soils

**DOI:** 10.3390/s20051336

**Published:** 2020-02-29

**Authors:** J.D. Jabro, W.B. Stevens, W.M. Iversen, B.L. Allen, U.M. Sainju

**Affiliations:** Northern Plains Agricultural Research Lab., ARS-USDA, 1500 N. Central Avenue Sidney, MT 59270, USA

**Keywords:** irrigation, soil moisture, soil moisture content, field capacity, permanent wilting point

## Abstract

Data-driven irrigation planning can optimize crop yield and reduce adverse impacts on surface and ground water quality. We evaluated an irrigation scheduling strategy based on soil matric potentials recorded by wireless Watermark (WM) sensors installed in sandy loam and clay loam soils and soil-water characteristic curve data. Five wireless WM nodes (IRROmesh) were installed at each location, where each node consisted of three WM sensors that were installed at 15, 30, and 60 cm depths in the crop rows. Soil moisture contents, at field capacity and permanent wilting points, were determined from soil-water characteristic curves and were approximately 23% and 11% for a sandy loam, and 35% and 17% for a clay loam, respectively. The field capacity level which occurs shortly after an irrigation event was considered the upper point of soil moisture content, and the lower point was the maximum soil water depletion level at 50% of plant available water capacity in the root zone, depending on crop type, root depth, growth stage and soil type. The lower thresholds of soil moisture content to trigger an irrigation event were 17% and 26% in the sandy loam and clay loam soils, respectively. The corresponding soil water potential readings from the WM sensors to initiate irrigation events were approximately 60 kPa and 105 kPa for sandy loam, and clay loam soils, respectively. Watermark sensors can be successfully used for irrigation scheduling by simply setting two levels of moisture content using soil-water characteristic curve data. Further, the wireless system can help farmers and irrigators monitor real-time moisture content in the soil root zone of their crops and determine irrigation scheduling remotely without time consuming, manual data logging and frequent visits to the field.

## 1. Introduction

Precise irrigation scheduling methods, based on smart soil moisture sensors, are needed to optimize crop production, enhance water use efficiency, minimize water loss, and reduce adverse environmental impacts. Under-irrigation can result in yield or quality reductions due to crop water stress. However, over-irrigation can have negative environmental consequences, including increased runoff, nitrate leaching, crop disease and increased water, fertilizer, time, labor and energy costs. 

Real-time monitoring soil moisture sensors have been used for a variety of agricultural and environmental applications, including water management and irrigation scheduling [1,2]. Watermark soil moisture sensors can provide continuous real-time measurements of water potential at various depths, within the soil profile, that can be used for irrigation scheduling. These sensors have been widely used for irrigation management and proven to be robust and reliable for measuring water potentials under a variety of field conditions [3,4,5,6]. 

Recently, Watermark sensors have been integrated into wireless capability for monitoring water potential in the soil profile from multiple sets of sensor nodes placed in the field and transmitting data from these nodes’ loggers to the web via radios and Cellular Gateway. This new wireless technology provides convenient ways for monitoring real-time soil moisture status in the root zone without frequent visits to the experimental site and the necessity to manually download the data from the loggers on a regular basis. 

Ref. [6] conducted a field study to evaluate real-time wireless smart sensors for scheduling irrigation in a cotton crop. They concluded that smart sensors offered true potential for accurately monitoring soil water status in soils and determined timing and amounts for real-time site specific irrigation applications. Ref. [7] deployed a successful wireless sensor network for irrigation management under maize in Malawi and evaluated the system in terms of cost, robust and performance.

Recently, Ref. [8] developed a method to implement soil water tension data from soil moisture sensors and water retention curve using the van Genuchten model to provide soil moisture status in the soil for irrigation scheduling and management. Their model was validated and incorporated into a web-based irrigation scheduling tool in conjunction with a wireless soil moisture sensing system to manage irrigation in large fields. Limited research has been published on the applications of wireless Watermark soil moisture sensors in conjunction with soil water characteristic curve for irrigation scheduling. Therefore, the objective of this study was to evaluate an irrigation scheduling method, based on soil matric potential, measured with wireless Watermark soil moisture sensors, which are installed in sandy loam and clay loam soils and soil-water characteristic curve data.

Watermark soil moisture sensors were selected in this study due to their simplicity, accuracy, ease of installation, and reduced labor intensity, maintenance, soil disturbance and relative affordability compared to other soil moisture sensors [4,5,6]. These characteristics, coupled with the wireless capability, make WM sensors more desirable for irrigation planning and management. 

## 2. Materials and Methods

### 2.1. Sites and Soils Description 

Field studies were conducted at two locations of different soil texture, one in North Dakota and the other in Montana. The North Dakota site is located at the North Dakota State University Nesson Valley Irrigation Research and Development Project (Nesson), approximately 37 km east of Williston, ND USA with latitude of 48:9:50 and longitude of 103:5:55. The soil is classified as Lihen sandy loam (sandy, mixed, frigid Entic Haplustoll) and consists of very deep, somewhat excessively or well drained, nearly level soil that formed in sandy alluvium, glacio-fluvial and eolian deposits [1,9]. The Montana site at the Montana State University Eastern Agricultural Research Center (EARC) is located approximately 2 km north of Sidney, MT USA with latitude of 47:43:32 and longitude of 104:9:5. The soil at EARC is classified as Savage clay loam (fine, smectitic, frigid Vertic Argiustolls), consists of deep, drained, nearly level and formed in alluvium parent material [1]. Particle size analysis and bulk density for both soils are given in Table 1. 

Irrigation amounts were applied as needed using a self-propelled overhead linear move sprinkler irrigation system (model 8000, Valmont Irrigation, Valley, NE, USA). Potential evapotranspiration (ET) values for sugarbeet as calculated by the Jensen-Haise model in 2017 growing season were 591 and 547 mm at the Nesson, and EARC research sites, respectively.

Sugarbeet was planted on 3–4 May, 21 April, 2017 and harvested on 25 September, 29 September, 2017 at the Nesson and EARC research locations, respectively.

### 2.2. Watermark Soil Moisture Sensor Description 

The WM soil moisture sensor is an electrical resistance device in a gypsum wafer surrounded by a granular matrix material, encased in perforated stainless steel sleeve. Water conditions, inside the WM sensor, change with corresponding variations in soil water conditions. These changes within the sensor are reflected by differences in electrical resistance between two electrodes imbedded within the granular matrix in the sensor. The two electrodes are located inside a plastic tube where the wires leads enter the sensor. Resistance between the electrodes decreases with increasing soil water content [1,4,9]. 

The WM sensors read soil moisture content as matric potential (soil water potential) in centibar (cbar), which is equivalent to kilopascal, kPa [4]. Soil matric potential is a negative pressure or tension, indicating how tightly water is held by the soil matrix under unsaturated conditions. It is usually expressed in negative pressure units, however for the purpose of this study, the negative sign was omitted to avoid any confusion in calculating the parameters in Campbell’s equation (Equations (3) and (5)). The water potential range of the WM sensor is from 0 to 200 cbar, with 0 cbar denoting a saturated soil and 200 cbar signifying a relatively dry soil. Soil water potential readings from WM sensors can be converted to volumetric water contents using charts or equations [4,9]. 

### 2.3. Description of IRROmesh Data Logging System 

**Figure 1:**
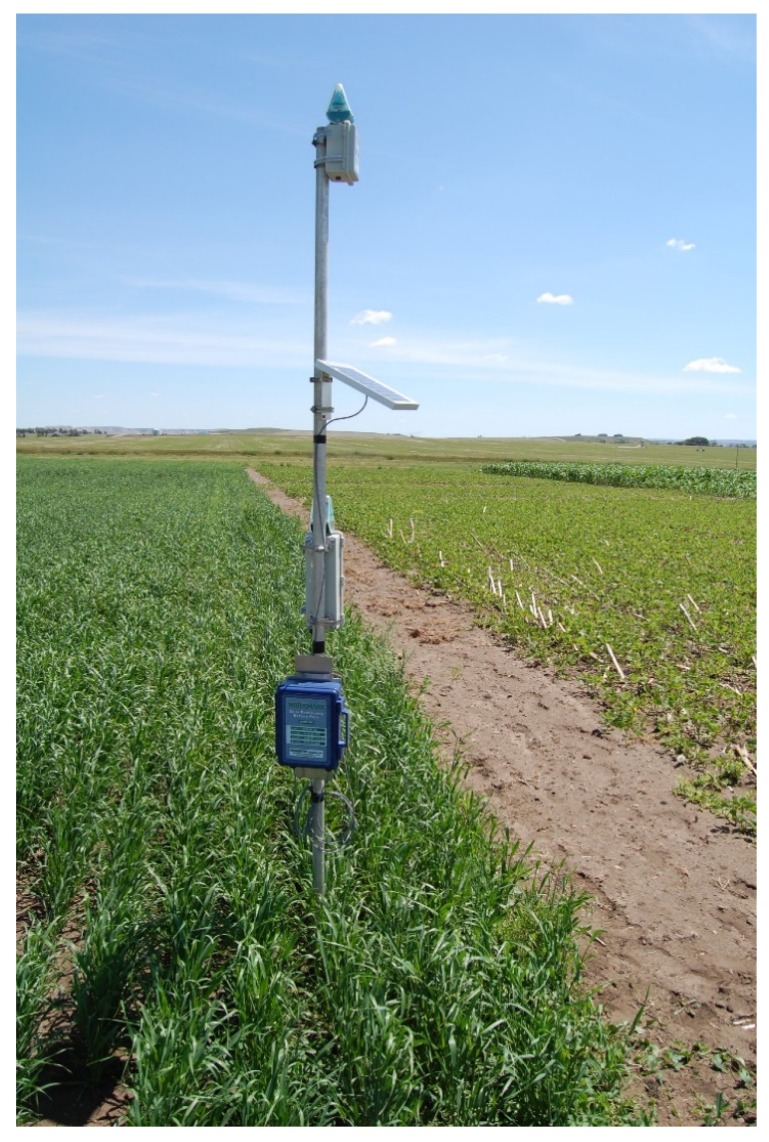
Watermark sensors (IRROmesh) wireless assembly in barley plots at the Nesson location.

The IRROmesh system uses 915 MHz or 868 MHz radios that are self-initializing and self-routing. The system is a wireless, solar-powered data-logging system that simplifies irrigation management using transmitter nodes that communicate with each other along a network that relays site-specific data (Figure 1). Each system node is capable of collecting data from three WM sensors and one temperature sensor, as well as a tipping bucket and a switch closure device, though we only used the WM and temperature sensors. Each node is self-contained with the radio, logging unit, charging unit, and antenna all built into the device (Figure 1). The logging node collects data, then transmits it to the Base Receiver Node (Model 975B). The Base Reciever Node transmits data to the Cellular Gateway (Model 975B). The Cellular Gateway sends data via a cellular modem to the web for viewing and final storage. The modem needs a SIM card configured for a cellular service provider (e.g, Verizon, AT and T) to facilitate connection to cellular towers. Data are collected even when the network appears to be down. The nodes transmit wirelessly real-time water potential measurements every 30 min to a remote host. Detailed information regarding IRROmesh system technology, WM sensors, installation and operation processes are available at [4]. 

IRRomesh nodes were installed in sugarbeet plots at each of the two study sites, three WM sensors wireless (Model 200SS, Irrometer Company, Inc. Riverside, CA, USA) were installed at soil depths of 15 cm, 30 cm, and 60 cm in the crop row between plants. Soil temperature sensors (Model 200TS) were installed in the soil above the WM sensors at 15 cm depth to correct WM electrical resistance output for variations in soil temperature of 21 °C. Soil water potential and temperature readings were continuously recorded using a data logger. 

### 2.4. Irrigation Scheduling 

Soil water potential values, measured at various depths, can be averaged using the weighted mean to reflect soil water contents in the crop rooting zone. In this study irrigation events were initiated when any of the depths’ WM readings approached the threshold level for a given crop at either location. However, we actually use crop water use (evapotranspiration, ET), visual evaluations of crop and soil conditions and the availability of irrigation water in addition to WM readings to determine when to irrigate. 

Using soil moisture content, at field capacity and permanent wilting point for soil, determined the available plant soil water capacity and maximum water depletion level, which was a trigger point for irrigation event scheduling. Presumably, the traditional value for irrigation is depletion of about 50% of plant available water capacity for most crops and soil types [10]. 

Field capacity is the amount of moisture in the soil after it has been thoroughly wetted and allowed to drain freely. Permanent wilting point is the amount of moisture in the soil when plants start to wilt and do not recover upon wetting [11,12,13]. Soil water content at field capacity levels for both soils was estimated in-situ using a soil-water characteristic curve [9]. Permanent wilting points for sandy loam and clay loam soils were obtained from literature and soil water characteristic curves [15]. Irrigation thresholds for WM sensors were established at the level of 50% of plant available water for both soils at each location. 

Soil volumetric water content (*θ*) from both sandy loam and clay loam soils were converted to matric water potential, ψ using a Campbell equation [9,14,15],

(1) $$\psi =\psi_{e}{\left(\frac{\theta }{\theta_{s}}\right)}^{-b}$$

where ψ is the soil matric potential, ψ_e_ is the air-entry potential of the soil, θ_s_ is the volumetric water content at saturation, and *b* is an empirical parameter determined from the soil-water retention curve [9,14,15]. Equation (1) can also be rearranged and solved in terms of *θ*: as:

(2) $$\theta =\theta_{s}{\left(\frac{\psi }{\psi_{e}}\right)}^{-1/b}$$

The estimated ψ_e_ and b parameters for a sandy loam were 1.637 kPa and 3.934, respectively, [9] and for a clay loam soil were 4.644 kPa, and 5.286, respectively, [15]. The θ_s_ values for each soil were estimated from soil bulk density and particle density data at each location and were 0.421 and 0.468 m^3^ m^–3^ for sandy loam, and clay loam soils, respectively.

The b parameter for a clay loam soil was estimated from the field capacity and permanent wilting point data [15]. as,

(3) $$b=\frac{\ln\psi_{PWP}-\ln\psi_{FC}}{\ln\theta_{FC}-\ln\theta_{PWP}}$$

where $\psi_{PWP}$ and $\psi_{FC}$ are soil water potentials at permanent wilting point (1500 kPa) and field capacity (33 kPa), respectively; $\theta_{FC}$ and $\theta_{PWP}$ are volumetric soil moisture contents (%) at field capacity, and permanent wilting point levels, respectively.

The air-entry potential value (ψ_e_) for a clay loam soil is estimated from Equation (4) as,

(4) $$\psi_{e}=\frac{a}{\theta_{s}^{b}}$$

where $\theta_{s}$ and b were previously defined for Equations (1)–(3), and a parameter was estimated as,

(5) $$a={\left(\frac{\theta_{FC}-\theta_{PWP}}{{\psi_{FC}}^{-1/b}-{\psi_{PWP}}^{-1/b}}\right)}^{b}$$

where $\theta_{FC}$ and $\theta_{PWP}$ are volumetric soil moisture content (m^3^ m^–3^) at field capacity and permanent wilting point, respectively; $\psi_{PWP}$ and $\psi_{FC}$ are soil water potential at permanent wilting point (1500 kPa), and field capacity (33 kPa), respectively [15]. 

The irrigation amounts can be calculated using WM sensors readings and soil water characteristic curve data as,

(6) $$d\;=\;\left(\theta_{FC}{-}_{\;}\;\theta_{50\%}\right)\times D$$

where d is the depth of water to be applied (irrigation amount) in cm, $\theta_{FC}$ is the soil moisture content at field capacity (upper point) in cm^3^ cm^–3^, $\theta_{50\%}$ is the soil moisture content at 50% of available water capacity (lower point or the maximum soil water depletion) in cm^3^ cm^–3^, and D is depth of plant root zone in cm. 

The irrigation amount (d) can be divided by the efficiency of the irrigation system to obtain accurate water application amounts. The efficiency of the mid-elevation spray application irrigation system at both locations was reported to be about 85% [16]. 

## 3. Results and Discussion

**Figure 2:**
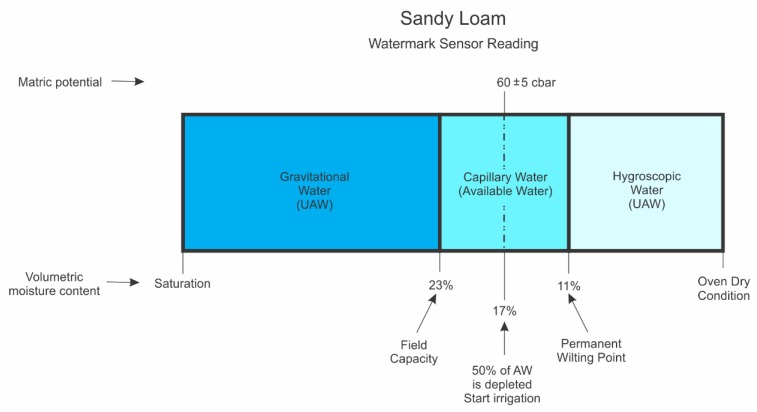
Schematic diagram of water types in the soil and soil water thresholds for a sandy loam soil. The AW and UAW denote available water, and unavailable water, respectively.

**Figure 3:**
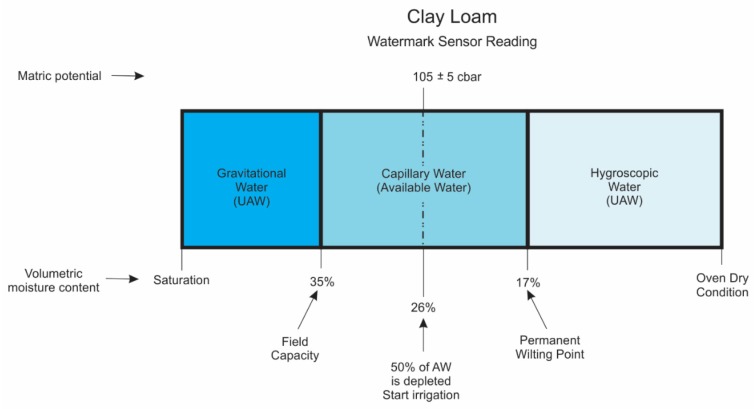
Schematic diagram of water types in the soil and soil-water thresholds for a clay loam soil. The AW and UAW denote available water and unavailable water, respectively.

There are three types of water present in the soil (i.e., gravitational water, capillary water, hygroscopic water) and soil water thresholds (i.e., saturation, field capacity, permanent wilting point, and oven dry condition). Water types and threshold values are given for both sandy loam and clay loam soils in Figure 2 and Figure 3, respectively. 

A soil is considered saturated when all pores (macropores and micropores) are filled with water and the soil matric potential approaches zero. The gravitational water moves through soil macropores by the action of gravity and is not available to the plant as it drains quickly; the capillary water is held in the soil micropores and moves via surface tension and capillarity; and the hygroscopic water is a thin layer of water surrounding soil particles that moves as water vapor. It is held tightly to soil particles by adhesion forces and is unavailable to the plant. The plant available soil water for plants is the difference between the moisture contents at field capacity and permanent wilting point, and both of these thresholds vary with soil texture and pore size distribution [11,12,13,15]. 

Based on in-situ measurements [9] and Campbell’s estimates [14,15], soil moisture content at field capacity and permanent wilting point were, respectively, about 23% and 11% for a sandy loam, and 35% and 17% for a clay loam (Figure 2 and Figure 3). Field capacity is the upper threshold of plant-available soil moisture that occurs directly after the irrigation or rainfall event. However, the lower trigger point of soil moisture content is at 50% of available soil moisture in the root zone, depending on soil texture, pore size distribution, crop type, root depth, growth stage and climatic conditions [1,17,18]. The available depleted soil moisture can be calculated as the difference between the moisture content at field capacity and current moisture content in the soil, divided by plant available soil moisture content in the root zone. 

Soil volumetric moisture content at 50% of available water capacity to trigger an irrigation event were 17% and 26%, for sandy loam, and clay loam soils, respectively (Figure 2 and Figure 3). The corresponding water potential readings (Equation (1)) from WM sensors to start an irrigation were approximately 60 ± 5 kPa and 105 ± 5 kPa for sandy loam, and clay loam soils, respectively. These soil water potential values suggest when an irrigation event should occur to refill the soil system (Figure 2 and Figure 3) before plants begin to wilt or show some signs of water stress.

**Figure 4:**
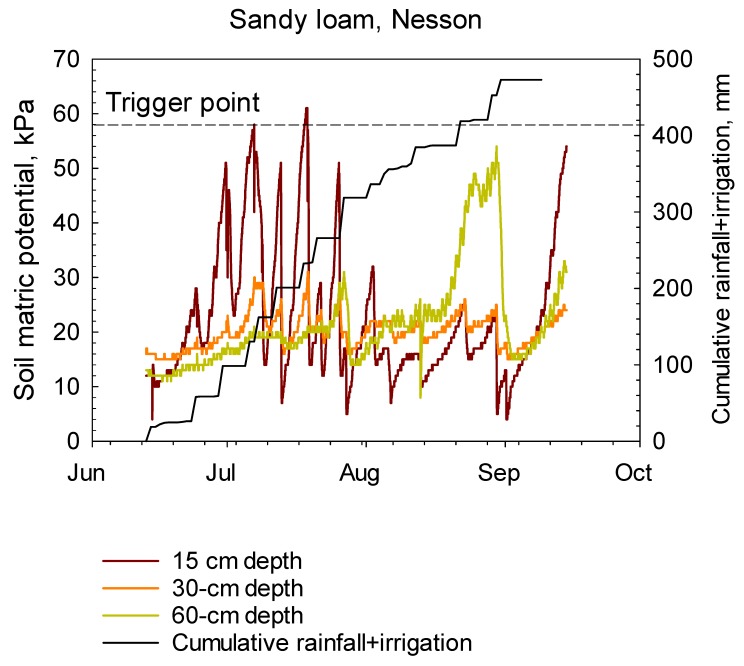
Hourly soil matric potentials from WM sensors at the 15, 30 and 60 cm soil depths, cumulative rainfall, and cumulative irrigation under sugarbeet during the 2017 growing season for a sandy loam soil at the Nesson site near Williston, ND.

**Figure 5:**
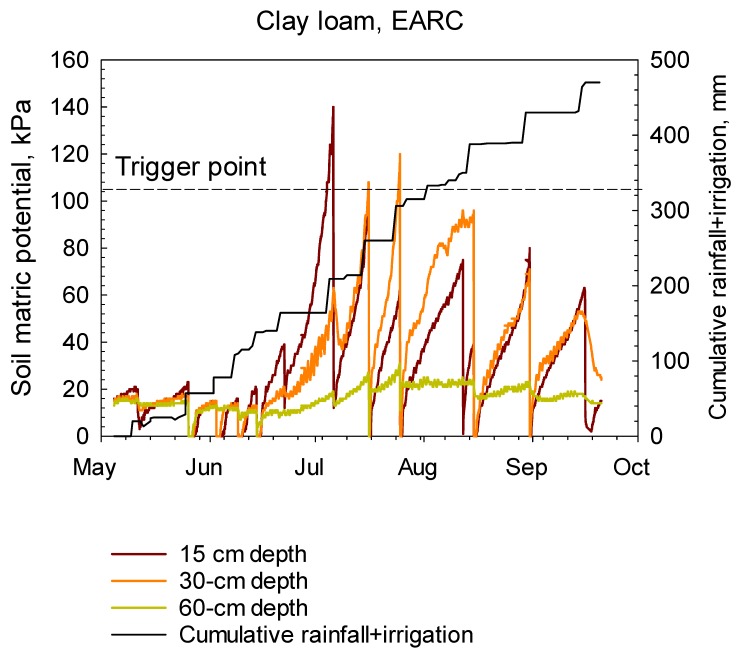
Hourly soil matric potentials from WM sensors at the 15, 30, and 60 cm soil depths, cumulative rainfall, and cumulative irrigation under sugarbeet during the 2017 growing season for a clay loam soil at the EARC site near Sidney, MT.

In this paper, two scenarios were introduced to present irrigation scheduling methods in sugarbeet plots for sandy loam and clay loam soils at the two locations. Real-time hourly soil matric potentials from WM sensors at the 15, 30 and 60 cm soil depths, cumulative rainfall, cumulative irrigation under sugarbeet during the 2017 growing season for sandy loam and clay loam soils are illustrated in Figure 4 and Figure 5, respectively. Soil matric potentials from WM readings in both soils decreased with irrigation and rainfall events. 

In a sandy loam soil at the Nesson location, soil matric potentials from a WM sensor placed at a 15-cm depth were generally maintained around, or below, the threshold level during the growing season, due to frequent irrigation, and rainfall events or rainfalls occasionally occurred shortly after irrigation events (Figure 4). Irrigation events were usually initiated when soil water potentials of the top depth’s sensor were near 58 kPa. However, WM readings showed that soil water potentials at the 30 cm and 60 cm depths were constantly below the threshold level (Figure 4). 

In a clay loam soil at the EARC location, irrigations were initiated when soil water potentials of the top depth’s sensor approached 105 kPa. Watermarks readings showed that soil water potentials at the 15, 30 cm and 60 cm depths were constantly below 105 kPa, except for the 15 cm and 30 cm depths between July 4–6, and July 24–26 periods, respectively, when WM readings exceeded the threshold level (Figure 5). This might be associated with an inadequate irrigation and lack of rainfall to refill these two zones during these periods of high crop water use. Soil water potentials at the 60 cm depth were continuously below 30 kPa during the entire growing season. 

To avoid any damage to crops due to water stress, it is better to irrigate at or preferably prior to reaching the maximum depletion level in the root zone. This level of soil moisture content is very critical and is considered the maximum available soil water to be removed from the root zone by plants before the next irrigation replenishment occurs. 

In some instances and depending on soil type, crop type, root depth and other factors, sandy soils may require a larger depletion limit (>50% of available water capacity) than clayey soils (<50%) of available water capacity because clayey soils generally hold water more tightly and plants exert more energy to absorb it than in sandy soils. 

This irrigation scheduling method can be used with both manual datalogging and wireless monitoring WM sensors. However, WM wireless sensors or an IRROmesh system can help farmers monitor real-time soil moisture content in the soil root zone of their crops and determine irrigation planning remotely. This irrigation management practice may enhance water use efficiency, sustain productivity, and increase net economic return, while maintaining environmental quality.

Our findings, coupled with previous research studies, indicate that soil moisture sensors have proven to be reliable and useful tools for measuring soil water potentials under various field conditions and scheduling irrigation more efficiently [3,5]. Our findings were consistent with those of [6,7] who reported that smart sensors have real potential to accurately monitor moisture content status in soils and determine irrigation management practices. 

## 4. Conclusions

The results showed that WM sensors provided practical tools for monitoring soil moisture status in the root-zone for irrigation scheduling in sugarbeet plots, for both sandy loam and clay loam soils at the two locations.

Whether data-logging is wireless or manual, WM sensors can be used to effectively monitor soil water potential status in the crop root zone and determine efficient irrigation events by simply setting two matric potential thresholds using soil water characteristic curve data. Each irrigation event can be initiated at the matric potential of soil water depletion level of 50% of plant available water capacity for sandy loam and clay loam soils. 

Wireless WM sensors can be used to monitor real-time soil moisture content in the root zone and determine irrigation planning remotely. This technology can provide convenient ways for continuously monitoring soil moisture conditions in the plant root zone without regular visits to the field and manually downloading data from the loggers. The system may enhance water use efficiency, improve crop yield, limit expenditure, and improve water quality. 

Mention of trade names, proprietary products, or specific equipment is intended for reader information only and constitutes neither a guarantee nor warranty by the ARS-USDA, nor does it imply approval of the product named to the exclusion of other products.

## Figures and Tables

**Table 1:** Soil particle size distribution and bulk density at 0–15, 15–30, and 30–60 cm depths for sandy loam and clay loam soils at Nesson (Williston, ND, USA) and EARC (Sidney, MT, USA) irrigated research sites.

Soil/Location	Depth cm	Sand	Silt %	Clay	Bulk Density (Mg m^–3^)
Sandy loam					
Nesson					
	0–15	66	17	17	1.56
	15–30	67	15	18	1.51
	30–60	75	12	13	1.60
Clay loam					
EARC					
	0–15	21	41	38	1.43
	15–30	20	43	37	1.39
	30–60	34	34	32	1.41
